# Multidimensional Approach Toward Spiritual Coping: Construction and Validation of the Spiritual Coping Questionnaire (SCQ)

**DOI:** 10.1007/s10943-014-9892-5

**Published:** 2014-06-05

**Authors:** Edyta Charzyńska

**Affiliations:** Department of Psychology, University of Social Sciences and Humanities, Chodakowska 19/31, 03-815 Warsaw, Poland

**Keywords:** Spiritual coping, Religious coping, Stress, Spirituality, Religiousness

## Abstract

The aim of the research was to construct the Spiritual Coping Questionnaire (SCQ). Two studies have been carried out: the first on the sample of 1,296 persons facing stressful situations, and the second, on 352 persons undergoing alcohol addiction therapy. The first study provided data for PCA and CFA, calculation of internal consistency, test–retest reliability and descriptive statistics of the questionnaire. The second study allowed the author to verify the construct and criterion validity of the tool. The final version of the SCQ is composed of 32 items constituting two scales: positive and negative spiritual coping. The scale of positive spiritual coping includes four subscales—domains (personal, social, environmental and religious), and the scale of negative spiritual coping, three subscales (personal, social and religious). The validity and reliability of the tool are satisfactory. The questionnaire can be used to measure spiritual coping, both among religious and non-religious people.

## Introduction

The increasing interest in research on spirituality results to a great extent from social changes occurring in the area of religious life: progressing secularization, increasing individualization and autonomization, growing faith in the possibility of achieving well-being in earthly life, psychologization of religion and the emergence of new spiritual trends (Luckmann 1967; Hill et al. 2000; Taylor 2007). Nowadays, limiting oneself only to studying religiousness, the ways of experiencing it and its functions, seems to overlook differences regarding the experiencing and development of spiritual life.

The interest in spirituality is also enhanced by empirical reports indicating its relationships with health and quality of life, emphasizing not only the cognitive value of deepening knowledge in the area but also the value of its application (Koenig 1994; George et al. 2000; Miller and Thoresen 2003).

### Spirituality Versus Religiousness

When raising the subject of spirituality, it is necessary to point out what assumptions concerning the relationships between spirituality and religiousness underlie the conducted research. Various approaches to the issue have been developed so far: Some of them treat both terms as identical, others emphasize the positive, personal character of spirituality and negative, institutionalized character of religiousness, and still others regard religiousness as a subset or superset of spirituality (Zinnbauer and Pargament 2005; Zinnbauer et al. 1999).

The approach adopted in this study assumes that the terms include both common and separate fields (Paloutzian and Park 2005; Emmons 1999). Religiousness is probably the most popular form of spiritual life, related with the human’s willingness to go beyond the material sphere. It may, however, include elements which are not directly related to spirituality, such as religious practices and activities connected with the religious community, especially when they are done habitually or when the overriding objective is to acquire mental and social comfort without a real bond with God/the Supreme Being (Socha 2000). On the other hand, religious life is not the only way to deepen the spiritual domain of life. People know various ways to develop their spirituality, e.g., by contemplating nature or art, strengthening their relationships with other people, or achieving self-transcendence through combating one’s own limitations and adversities.

### Definition of Spirituality and Its Domains

Spirituality can be defined as an attribute of every human, a constitutive trait one has from birth to death (McCarroll et al. 2005). It has a dynamic character: It can develop, change forms or become invisible (Heszen-Niejodek and Gruszczyńska 2004). Some authors, e.g., Piedmont (1999), ascribe a special role to it, suggesting it should be considered as the sixth domain of personality.

Despite definition difficulties, researchers try to indicate the components of spirituality. In many definitions, transcendence is the core element (Chiu et al. 2004; Martsolf and Mickey 1998; Lewis 2008). It is defined as the activity of going beyond or above the “self” (in the meaning of growth and development; Heszen-Niejodek and Gruszczyńska 2004).

The relational character is often ascribed to spirituality (Martsolf and Mickey 1998; Ross 2006; Lewis 2008). The object of transcendence can be: the person themselves (personal domain) or an external object—the Supreme Being/God (religious domain), another person (social domain) or the Universe/nature (environmental domain; Hill et al. 2000; Fisher 2011).

In this paper, it is assumed that the concept of transcendence is the core of spirituality, which, in turn, is treated in a dynamic and relational way, considered in four dimensions: personal, social, environmental and religious ones. Such an approach does not eliminate traditionally understood religiousness from spirituality but at the same time it supplements spirituality with non-religious aspects, sometimes called “humanistic” ones (Newby 1996).

### Religious Coping

The most well-known theory concerning the coping with stressful situations with the use of religious resources was formulated by Pargament (1997). In it, Pargament referred to the transactional model of stress, assuming that an individual plays an active role in interpreting and reacting to life stressors (Lazarus and Folkman 1984). Pargament (1997) defines religious coping as efforts to understand and deal with life stressors in ways related to the sacred, whereas the word “sacred” does not only refer to the traditionally understood God, divinity or the Supreme Power but also to other aspects of life which are related to divinity or have divine qualities. One of the main goals of his theory was to indicate and describe the methods of religious coping understood as situational expressions of an individual’s religiousness oriented at the search of meaning. On the basis of research, two kinds of religious coping have been distinguished: positive and negative one (Pargament et al. 1998, 2000). Positive coping is based on solid, trustful and safe relation with God, whereas in the case of negative religious coping, the individual displays a less safe and distrustful relation with God and carries on spiritual fight. Generally, research confirms the beneficial health impact of positive religious coping strategies and detrimental impact of negative religious coping strategies (Pargament et al. 1998, 2003; McConnell et al. 2006).

### Spiritual Coping

Although the terms “religiousness” and “spirituality” are differentiated in the literature, the division is still not common in the stress field. Most publications apply the term “religious/spiritual coping” (Thuné et al. 2006; Klaassen et al. 2006). Most research tools only measure religious coping. Few scales take into consideration the spiritual, non-religious methods of coping (Baldacchino and Buhagiar 2003). Hence, there is a need to intensify efforts on spiritual coping and seek its relationships with health.

Analogically to the definition of religious coping proposed by Pargament (1997), spiritual coping with stress can be defined as attempts to overcome the stressor on the basis of what is transcendent. Transcendence can assume different directions: self-improvement of oneself, deepening relations with others, building the sense of unity with nature or attachment to and trust in the Divine Being (Miller and Thoresen 2003; Hill et al. 2000). The definition takes transcendence into account as the core of spirituality and at the same time emphasizes its multidimensionality.

As already mentioned, Pargament et al. (2003) are aware that religiousness has its “dark side,” which must be taken into consideration in research and clinical practice. It may be supposed the situation is similar in the case of spirituality. However, the thing is not to assume the division into “positive spirituality” and “negative spirituality.” The concept of negative spirituality is promoted by parareligious trends, but it seems to be unacceptable in scientific discourse due to lack of clear definitions and the difficulty with operationalization. Hence, it seems more appropriate to treat spirituality as a whole, whose manifestations can adopt different forms. That is what happens in the case of positive and negative spiritual coping.

Positive spiritual coping would then involve taking cognitive and behavioral efforts aimed at solving a difficult situation, which—depending on the domain—are manifested in:

1. pursuit of a goal, sense and meaning, concentration on one’s internal life, attempts to overcome one’s weaknesses and acquiring more and more self-knowledge, looking for internal peace and harmony (the personal domain);
2. establishing and maintaining deep and valuable relations with other people, heeding moral values, treating people fair, caring about others, willingness to help, displaying love, empathy and compassion (the social domain);
3. concentration on the sense of attachment and belonging to nature, perceiving harmony and order in it, treating nature as friendly to humans, openness to noticing miracles in nature (the environmental domain);
4. maintaining solid relation with God/the Supreme Power, based on the sense of presence, love and trust (the religious domain).

Negative spiritual coping makes it impossible for an individual to draw strength from spiritual resources, blocks the pursuit of sense and meaning in life, hinders its growth, “upward movement” and going beyond what is material. It may manifest itself in various forms:

1. negating the goal and meaning of one’s life, emphasizing one’s weaknesses and limitations, concentration on one’s transgressions (the personal domain);
2. perceiving people as inherently egoistic and caring only about their interests, which results in aversion, hostility or envy toward others, blocking the possibility to establish and maintain deep, valuable interpersonal relations (the social domain);
3. treating nature as hostile to humans and posing threat, emphasizing human helplessness and insignificance in the face of the laws of nature (the environmental domain);
4. internal religious fight displayed in holding a grudge toward God/the Supreme Power, blaming Him/It for one’s own failures, negating His/Its love and care for humans.

On the basis of research on religious coping (Pargament et al. 2011), it is assumed that the two kinds of spiritual coping are relatively independent of one another: A person may use positive and negative religious coping simultaneously.

Extending the research on coping with stress by spiritual, not only religious, aspects, is significant for both theoretical and practical reasons. On the one hand, the construction of a proper tool to measure spiritual coping with stress would allow researchers to perform multi-aspect measurement of relationships between spiritual coping strategies and functioning, and on the other hand, it would constitute the basis for measuring the effectiveness of therapeutic activities considering spirituality as a potential resource in coping with various stressors.

The aim of the presented two studies was a construction, and preliminary validation of a questionnaire designed to measure spiritual coping, based on definitions of spirituality referring to the concept of transcendence, indicating the multidimensionality of spirituality, as well as on the theory of coping with stress by Lazarus and Folkman (1984) and the concept of religious coping by Pargament (1997).

## Study 1

### Methods

#### Participants and Procedure

Study 1, which helped develop the final version of the tool, was conducted between March 2012 and April 2013 in the Silesia Province in Poland. The research sample was purposefully selected. It included people assumed to be facing stressful situations: people in hospitals and undergoing treatment in rehabilitation centers (patients suffering from cancer or those who had had a heart attack), people in addiction treatment centers, caring for ill family members, after a divorce or breakdown of a close relationship and people who were unemployed or facing the risk of losing their job. The research team contacted institutions which had access to potential respondents. 1,516 adults were asked to participate in the study, and 1,308 (86.3 %) agreed to do so. The participants were informed about the anonymous and voluntary nature of participation and then signed a consent form. Each respondent was asked to return the questionnaire within a week. No incentive was offered for participating. In the end, properly filled-out paper-and-pencil questionnaires were provided by 1,296 persons (see Table 1).

**Table 1:** Socio-demographic characteristics of the sample (Study 1)

	*N* = 1,296	%
Sex		
Women	654	50.5
Men	640	49.4
N/A	2	.1
Age		
18–28	436	33.6
29–39	298	23.0
40–50	293	22.6
51–61	178	13.8
62–72	58	4.5
73–83	24	1.8
N/A	9	.7
Education		
Elementary	42	3.3
Lower secondary	31	2.4
Vocational	213	16.4
Secondary	570	44.0
Higher	428	33.0
N/A	12	.9
Place of residence		
Village	197	15.2
Town up to 100 thousand residents	448	34.6
Town between 100 and 500 thousand residents	567	43.8
Town over 500 thousand residents	73	5.6
N/A	11	.8

Based on the respondents’ answers, the following categories of stressful situations were singled out: own bodily or mental illness—482 persons (37.2 %), illness of a loved one—89 persons (6.9 %), death of a loved one—71 persons (5.5 %), breakdown of an important relationship—276 persons (21.3 %), financial problems—292 persons (22.5 %), work problems—86 persons (6.6 %).

So as to calculate the test–retest reliability of the questionnaire, 300 people randomly selected from the male and female groups separately (150 of each group) were asked to fill in the questionnaire again 6 weeks later. The questionnaires were returned by 248 respondents (82.7 %), 143 women and 105 men.

#### Measures

##### Spiritual Coping

Step 1. The Spiritual Coping Questionnaire (SCQ) was constructed to measure spiritual coping. With the use of literature concerning psychology, philosophy and religion, the author prepared a set of 120 items which referred to four aspects of spiritual coping based on the dimensions of transcendence: personal, social, environmental and religious. Two aspects of spiritual coping were singled out: the positive one and the negative one. Eight scales were prepared, each including 15 items, 4 of which referred to positive spiritual coping and 4, to the negative one. The 5-point Likert scale was used with the rates: 1—“very inaccurately,” 2—“rather inaccurately,” 3—“neither inaccurately nor accurately,” 4—“rather accurately,” 5—“very accurately.”

The following instruction was prepared:

The statements presented below refer to different ways of coping with difficult life events. If you are currently in a hard situation, please describe briefly what it is related to. It may be e.g. an illness, a breakdown of a close relationship, financial problems etc. Please indicate how well each of the statements describes what you did in the past four weeks when dealing with this stressful situation. Keep in mind that the way of coping involves different methods of confronting a problem; not always do they lead to a positive solution. What is important for the purpose of this survey is whether and how often you have used a given way of coping, not the results it brought.

Step 2. The questionnaire was subject to linguistic proofreading by a Polish linguist and then evaluated by five competent independent judges—psychologists, academics knowledgeable about the process of construction and validation of psychological tools. Their tasks were to assess: (a) the clarity and validity of instructions, (b) the clarity and validity of particular items, (c) the consistency of items with the assumed factor structure of the tool, and (d) to provide any additional comments to improve the content and layout of the tool. The judges were given the questionnaire and presented the conceptualization of the domains and aspects of spiritual coping. All the assessments were done with the use of a 5-point Likert scale, in which 1—“completely unclear/invalid,” and 5—“completely clear/valid.”

Step 3. Two rates given by the judges helped choose the items for the experimental version of the tool: the rate of clarity of particular items and the rate of the items’ consistency with the assumed factor structure of the questionnaire. The concordance among the competent judges, expressed with Kendall’s W coefficient, was satisfactory: .78 in the case of item clarity and .71 in the case of consistency with the assumed factor structure. The items which were evaluated as most unclear and/or insufficiently representing the assumed scale of the tool were removed from further analyses. The items which received the average rate of at least 4.0 both in the case of clarity and consistency with the assumed factor structure were included in the experimental version of the tool. The judges’ comments regarding the graphic layout of the tool were also taken into consideration. Finally, the experimental version of the questionnaire was composed of 80 items, and each scale included 10 items rated on a 5-point Likert scale.

##### Analyses

All the calculations were done with the use of SPSS and AMOS version 20.0 software. The data obtained from half of the cases (648) were used to conduct principal component analysis (PCA), and the other half, to check the model-to-data goodness of fit with the use of confirmatory factor analysis (CFA). The descriptive statistics and internal consistency indices were calculated on the basis of data obtained from the whole sample. Gender differences in mean scores were tested for statistical significance using a *t* test. The test–retest reliability was calculated with the use of correlation coefficients.

### Results

#### Exploratory Analysis

The Bartlett’s test and Kaiser–Mayer Olkin measure were applied so as to verify the legitimacy of conducting PCA. The Bartlett’s test showed the significance at the level of *p* < .001 (*χ* ^2^(496) = 11,370.54), and the KMO index was .933. On the basis of these results, PCA was carried out in order to reduce the number of items.

Oblimin rotation was chosen, because correlations between the subscales of the tool were expected. A scree plot and additionally the Kaiser criterion were used to determine the number of components. Both methods suggested the existence of eight components, jointly accounting for 53.75 % of the variance of the “spiritual coping” variable. Further, the pattern matrix and structure matrix were analyzed. The items which (a) loaded their own factors at the level lower than .4, or (b) loaded more than one factor at the same time, were removed from the questionnaire. The version of the questionnaire reduced as a result of the analyses included 36 items.

After the PCA, correlations between the questionnaire subscales were analyzed. Generally, they correlated with each other as expected: the subscales representing positive spiritual coping correlated positively with each other, so did the subscales measuring negative spiritual coping. What is more, negative correlations or no correlations were observed between the subscales measuring positive and negative spiritual coping (see Table 2). A weak negative correlation was found between positive and negative spiritual coping among women (*r* = −.13, *p* = .009) and men (*r* = −.10, *p* = .013). Surprising results, contrary to hypotheses, were found in the case of negative environmental spiritual coping (see again Table 2). That domain correlated positively with all the subscales of the questionnaire, most strongly with positive environmental coping (*r* = .42; *p* < .001). It indicated the non-specific character of the negative environmental coping subscale: The persons who used other spiritual coping strategies used negative environmental coping as well, while those who did not use the other spiritual coping strategies did not resort to negative environmental coping either. After the assessment of advantages and disadvantages of retaining that subscale in the final version of the tool, in the end it was removed.

**Table 2:** Correlations between the scales and subscales of the SCQ

	Spiritual coping									
	Positive					Negative				
	General	Personal	Social	Environmental	Religious	General	Personal	Social	Environmental	Religious
pos_general	1	.69***	.81***	.77***	.82***	−.10*	−.12**	−.08*	.20***	.01
pos_personal	.59***	1	.57***	.41***	.43***	−.08*	−.05	−.09*	.12**	−.04
pos_social	.81***	.49***	1	.56***	.48***	−.09*	−.05	−.12**	.12**	−.04
pos_environ	.73***	.27***	.50***	1	.42***	−.02	−.08*	.03	.41***	.03
pos_religious	.82***	.32***	.52***	.37***	1	−.11**	−.16***	−.08*	.20***	.03
neg_general	−.13**	−.13**	−.17***	−.05	−.17***	1	.84***	.73***	.29***	.71***
neg_personal	−.16***	−.10*	−.11**	−.12**	−.14***	.84***	1	.34***	.21***	.45***
neg_social	−.11**	−.10*	−.17***	.05	−.14**	.73***	.36***	1	.23***	.35***
neg_environ	.23***	.14***	.13**	.43***	.22***	.31***	.26***	.26***	1	.20***
neg_religious	−.12**	−.10*	−.11**	−.05	−.11**	.76***	.51***	.36***	.23***	1

*N* = 1,294; women = 654, men = 640; * *p* < .05; ** *p* < .01; *** *p* < .001. The upper part of the matrix shows the values of the correlation coefficients for males, the lower refers to females

The negative environmental coping subscale was eventually removed from the SCQ

After the removal of the negative environmental coping subscale, the final version of the tool included 32 items. They were then subjected to PCA with Oblimin rotation. As expected, the scree plot and Kaiser criterion showed the legitimacy of extracting seven components, jointly explaining 67.52 % of variance of the “spiritual coping” variable. All the items in the questionnaire loaded their factors at least at the level of .4, at the same time loading others at the level lower than .4 (Table 3).

**Table 3:** Pattern matrix from the PCA in Study 1

Item no.	Spiritual coping						
	Positive				Negative		
	Personal	Social	Environmental	Religious	Personal	Social	Religious
27	.794						
31	.697						
2	.683						
6	.653						
26		.844					
17		.757					
32		.752					
11		.734					
22		.648					
29		.512					
20			.887				
7			.864				
28			.816				
23			.810				
1			.793				
4				.889			
13				.867			
5				.866			
30				.864			
25				.860			
9				.832			
10					.858		
24					.851		
21					.794		
19					.740		
14						.866	
12						.782	
8						.702	
16						.623	
15							.904
3							.813
18							.581

The table presents 7-component solution, after the removal of negative environmental coping subscale. Only loadings >.4 are presented. *N* = 648

#### Confirmatory Factor Analysis

**Fig. 1:**
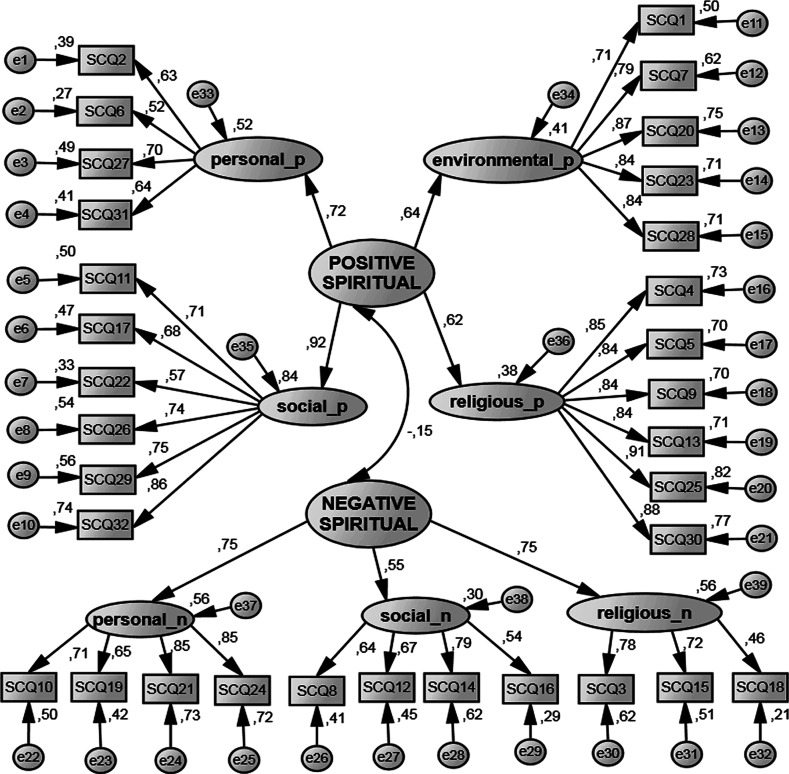
Confirmatory factor analysis results. SCQ1, SCQ2… mean the consecutive items of the SCQ. “Personal_p,” “social_p,” “environmental_p” and “religious_p” stand for positive spiritual coping in a particular domain, whereas “personal_n,” “social_n” and “religious_n” mean negative spiritual coping in a specific domain. *N* = 642

With the use of CFA, the model-to-data goodness of fit was checked. Apart from seven first-order factors, the existence of two second-order factors was assumed: positive (made up of four first-order factors) and negative (made up of three first-order factors) spiritual coping (Fig. 1). Before conducting the calculations, the assumptions of CFA were verified. Box plots were made, and the Mahalanobis distance was calculated. Six outliers were removed. Since missing data proved to be missing completely at random, the data were imputed with the expectation–maximization imputation (EM) method.

The good fit of the model to data is proved among others by statistical insignificance of the *χ* ^2^ test. The value of that statistic was 1,101.95 (456, *N* = 642) and was statistically significant at the level of *p* < .001. When interpreting the value of *χ* ^2^ statistic, it should be remembered that it is strongly dependent on the size of the sample and statistical significance is usually achieved when the number is high (over 400), which increases the risk of type II error (Gatignon 2010). Therefore, obtaining a significant result in the *χ* ^2^ test does not prove that the model does not fit the data.

The appropriate indices are more reliable to measure the goodness of fit. Therefore, the Normed Fit Index (NFI), Tucker–Lewis Index (TLI), Comparative Fit Index (CFI) and root mean square error of approximation (RMSEA) were calculated. A model can be considered as well fitting the data when NFI, TLI and CFI > .95, and RMSEA < .06 for continuous variables (Hu and Bentler 1999).

The indices had the following values, respectively: NFI = .924, TLI = .946, CFI = .951, RMSEA = .047. The obtained index values are close to the required ones, so the model can the regarded as fitting the data well.

#### Correlations Between Spiritual Coping and Socio-demographic Variables

On the basis of studies on religiousness and spirituality (Davie and Vincent 1998; Atchley 2009), a positive correlation between age and positive spiritual coping was expected. The relationship was indeed confirmed (*r* = .20; *p* < .001). In addition, a correlation was noted between spiritual coping and education: the higher the education one had, the less frequently they resorted to negative spiritual coping (*r* = −.19; *p* < .001).

Gender differences concerning spiritual coping were also calculated (Table 4). As regards women, a higher level of positive personal (*p* = .029) and religious (*p* = .001) coping was noted, whereas in the case of positive environmental coping (*p* = .006) and negative personal coping, the results were lower (*p* = .008) in this group as compared to men.

**Table 4:** Gender differences and reliability of the scales and subscales of the SCQ

Aspects of spiritual coping	*M* for women	SD for women	*M* for men	SD for men	*t*	*p*	Internal consistency (*α*)	Test–retest reliability	Sample item
Positive_general	3.36	.77	3.29	.85	1.57	>.05	.92	.78	
Positive_personal	3.64	.79	3.54	.83	2.19	.029	.79	.64	I was trying to find inner peace within myself
Positive_social	3.80	.80	3.72	.87	1.88	.060	.87	.72	I was nurturing my attitude of love toward other people
Positive_environm.	2.82	1.12	3.00	1.20	−2.74	.006	.92	.73	I was seeking closeness to Nature
Positive_religious	3.17	1.26	2.92	1.35	3.35	.001	.95	.82	I was turning to God/Higher Being with every matter important for me
Negative_general	1.89	.72	1.95	.73	−1.50	>.05	.82	.72	
Negative_personal	1.92	1.00	2.07	1.10	−2.64	.008	.77	.65	I was convincing myself that my life had no goal whatsoever
Negative_social	1.94	.84	2.02	.88	−1.65	.099	.79	.75	I was trying to prove to other people that they were egoists
Negative_environm.	2.01	.70	1.99	.79	1.28	>.05	.79	.72	I was thinking that nature threatened people
Negative_religious	1.78	.90	1.69	.84	1.85	.065	.67	.68	I was accusing God/Higher Being of what happened in my life

Descriptive statistics: *N* = 1,294; women = 654, men = 640; internal consistency: *N* = 1,296; test–retest reliability: *N* = 248

The negative environmental coping subscale was finally removed from the SCQ

In both sexes, the level of positive spiritual coping was higher than the negative one, which is in agreement with the results of studies on religious coping (Zwingmann et al. 2006; Bearon and Koenig 1990).

#### Reliability

All the scales and subscales of the SCQ had good or satisfactory reliability measured with the internal consistency coefficient Cronbach’s *α* (*α* = .67–.95). Reliability of the positive spiritual coping scale was *α* = .92, and of the negative spiritual coping scale, *α* = .82. Stability of the SCQ calculated with the test–retest method (a 6-week interval) also proved to be satisfactory: the correlation coefficient was *r* = .78 for the positive spiritual coping scale, and *r* = .72 for the negative spiritual coping scale (see again Table 4).

## Study 2

Study 2 was aimed at verifying the construct and criterion validity of the SCQ. As regards construct validity, correlations between spiritual coping and the related constructs were tested. Spiritual coping is understood as a manifestation of an individual’s spirituality which can be activated in a stressful situation. That definition led to hypotheses concerning positive relationships between positive spiritual coping and spirituality including its domains as well as negative relationships between negative spiritual coping and the above-mentioned variables. In addition, correlations between spiritual and religious coping as well as relationships between spiritual coping, gratitude and forgiveness were studied.

As already mentioned, the research indicates significant relationships between religious coping and the mental and physical health. In order to verify the criterion validity of the SCQ, correlations between spiritual coping and mental and physical functioning were estimated, after controlling for religious coping. On the basis of literature review (Pargament et al. 2011), stronger relationships of spiritual coping with mental health were expected than with physical health. Moreover, stronger correlations between negative coping strategies and physical and mental functioning were hypothesized than in the case of positive strategies.

### Methods

#### Participants and Procedure

The study involved 352 persons (257 men and 95 women) addicted to alcohol, entering a short-group therapy in outpatient wards. The mean age was *M* = 41.42 years, SD = 10.14, and the mean duration of addiction, *M* = 14.24 years, SD = 8.41. Every participant signed a consent form. No incentives were used. The respondents were accompanied by trained students when doing the questionnaires.

#### Measures

Apart from measuring spiritual coping with the SCQ, the following variables and tools were used in the study.

#### Spirituality

The Self-Description Questionnaire by Heszen-Niejodek and Gruszczyńska (2004) was used to measure spirituality. The questionnaire includes 20 items evaluated on the 1–5 scale, making three subscales: harmony, ethical sensitivity and religious attitude. Adding up the results obtained in the three subscales provides the general spirituality level. The questionnaire has satisfactory psychometric properties: reliability measured with the test–retest method was .88. Construct validity has been confirmed by significant correlations between the questionnaire and other scales measuring similar constructs.

#### Religious Coping

The Brief RCOPE (Pargament et al. 1998) adapted into Polish by Jarosz (2011), was used to measure religious coping. It is a 14-item tool composed of two scales: positive and negative religious coping. The items are evaluated on a 4-point Likert scale. The questionnaire is often used in scientific studies, among others due to the small number of items, which is its advantage.

#### Gratitude

The Polish adaptation of the GQ-6 (McCullough et al. 2002) by Kossakowska and Kwiatek (2012) was used to test the level of gratitude. The questionnaire is made up of 6 items evaluated on a 7-point Likert scale. The questionnaire’s psychometric properties are acceptable, and the reliability index, satisfactory (.72). Confirmatory analysis proved the relative goodness of fit of Polish data to the original one-factor tool structure.

#### Forgiveness

The indices developed by Toussaint et al. (2001), in the Polish adaptation by Charzyńska and Heszen (2013), were used to measure forgiveness. The Polish version of the tool is made up of three subscales: self-forgiveness, forgiveness of others and feeling forgiven by God, constituting the general scale of forgiveness. The tool’s structure has been confirmed by EFA and CFA. The scale’s reliability is satisfactory and ranges from .65 to .91. The construct validity and criterion validity of the tool have been confirmed.

#### Physical and Mental Well-Being

The Polish adaptation of the 36v2 Health Survey Questionnaire (Ware et al. 2000) by Żołnierczyk-Zreda et al. (2009) was used to measure the quality of life. The questionnaire includes 36 questions grouped into eight subscales (physical functioning, role-physical, bodily pain, general health, vitality, social functioning, role-emotional and mental health), giving two summary indices: physical health and mental health. The reliability of particular subscales measured with the internal consistency coefficient Cronbach’s *α* is good or satisfactory for most subscales (.73–.96); the criterion validity of the tool is also satisfactory.

### Results

The relationship between positive and negative spiritual coping was insignificant (*r* = −.052; *p* > .05). Positive spiritual methods of coping were positively correlated with spirituality (*r* = .28–.68), positive religious coping (*r* = .27–.83), gratitude (*r* = .22–.42), and forgiveness (*r* = .24–.39). Negative spiritual coping was negatively related to all the above-mentioned variables. Furthermore, positive spiritual coping was negatively correlated with negative religious coping (*r* = −.10 to −.26). As expected, positive relationships between negative spiritual coping and negative religious coping were also noted (*r* = .34–.44). To conclude, the obtained results supported the construct validity of the SCQ (see Table 5).

**Table 5:** Conctruct validity of the SCQ

	Spiritual coping								
	Positive					Negative			
	General	Personal	Social	Environmental	Religious	General	Personal	Social	Religious
Spirituality_general	.62***	.30***	.56***	.28***	.68***	−.21**	−.39***	−.34***	.34***
Inner harmony	.42***	.28***	.47***	.08	.26***	−.26***	−.34***	−.31***	−.10
Ethical sensitivity	.67***	.45***	.66***	.13*	.54***	−.13*	−.19***	−.35***	.06
Religious attitude	.48***	.14*	.37***	.01	.79***	−.15*	−.40***	−.24***	.18**
Positive religious coping	.58***	.34***	.45***	.27***	.83***	−.19**	−.16*	−.20**	−.14*
Negative religious coping	−.19**	−.10	−.17**	−.15**	−.26***	.44***	.38***	.34***	.40***
Gratitude	.42***	.33***	.22***	.26***	.25***	−.19**	−.21**	−.15**	−.16**
Forgiveness_general	.39***	.24***	.27***	.28***	.37***	−.35***	−.38***	−.24***	−.21***
Self-forgiveness	.01	−.01	.02	.07	.03	−.34***	−.31***	−.22***	−.27***
Forgiveness of others	.25***	.19***	.25***	.23***	.15*	−.34***	−.25***	−.38***	−.22***
Feeling forgiveness by God	.46***	.22***	.24***	.26***	.58***	−.14*	−.20***	−.08	.04

*N* = 352; * *p* < .05; ** *p* < .01; *** *p* < .001

The relationships between spiritual coping and mental and physical health were calculated with partial correlation coefficients. After controlling for positive and negative religious coping, three subscales of the SCQ were correlated with physical functioning: positive spiritual coping in the social domain (*r* = −.12; *p* = .026), negative spiritual coping in the personal domain (*r* = −.26; *p* < .001) and negative spiritual coping in the social domain (*r* = −.15; *p* = .005). Furthermore, the results confirmed that after controlling for positive and negative religious coping, all additional (non-religious) spiritual coping subscales were correlated with mental functioning. Precisely, mental functioning correlated positively with: positive spiritual coping in the personal (*r* = .16; *p* = .003), social (*r* = .23; *p* < .001) and environmental (*r* = .21; *p* < .001) domains, whereas it was negatively related to negative spiritual coping in the personal (*r* = −.46; *p* < .001) and social (*r* = −.20; *p* < .001) domains. These results proved that spiritual but non-religious coping was an independent component of physical and mental functioning.

As can be seen from the above results, stronger relationships of spiritual coping with mental health than with physical health were noted. Moreover, negative spiritual coping was negatively correlated both with mental and with physical functioning, while positive spiritual coping correlated positively almost exclusively with mental functioning.

## Discussion

The SCQ approaches spirituality and spiritual coping from the broad perspective. It allows us to obtain both general and specific results regarding positive and negative spiritual coping. General results are obtained by averaging the results received in particular subscales—four (personal, social, environmental and religious) in the case of positive spiritual coping with stress and three (personal, social and religious) in the case of negative spiritual coping. The results of particular subscales are obtained by averaging the responses to the appropriate items of the questionnaire.

Good or satisfactory psychometric properties of the tool were confirmed, both regarding its reliability and validity. The obtained results indicate relative independence of positive and negative spiritual coping. Hence, it is possible that a person uses both positive and negative coping strategies in a stressful situation. That result is additionally supported by studies on religious coping, indicating the orthogonality of positive and negative religious coping (Pargament et al. 2011).

However, the assumed structure of the tool was not confirmed in full. After analyzing matrices of correlations between the scales of the questionnaire, the scale of negative environmental coping was removed. It correlated positively with all the remaining subscales, most strongly with positive environmental coping, which suggested it was non-specific. It is impossible to rule out that the effect results from the study sample and cultural specificity. Therefore, when adapting the tool, it would be recommended to compare the current 7-subscale version of the tool with an 8-subscale version, taking into consideration the subscale of negative environmental coping.

The SCQ is designed to carry out research among adults and young adults. The time of doing the questionnaire is approximately 5–10 min. It is a relatively brief tool which allows us to get the view of the applied strategies of spiritual coping in the face of a stressful situation both among religious and non-religious people. Its unquestionable advantage is that it takes into consideration many domains of spiritual coping and singles out its positive and negative aspects. It allows a more profound look at spirituality and different variants of its expression in a stressful situation. The study of relationships between spiritual coping and mental and physical functioning proved the utility of additional (non-religious) coping scales, suggesting that the SCQ may be a valuable supplement to the existing tools used for studying religious coping. Further studies on the tool’s validity are recommended, including the possibility of changes in spiritual coping with stress as a result of treatment.
